# SUPPORT Tools for evidence-informed health Policymaking (STP) 9: Assessing the applicability of the findings of a systematic review

**DOI:** 10.1186/1478-4505-7-S1-S9

**Published:** 2009-12-16

**Authors:** John N Lavis, Andrew D Oxman, Nathan M Souza, Simon Lewin, Russell L Gruen, Atle Fretheim

**Affiliations:** 1 Centre for Health Economics and Policy Analysis, Department of Clinical Epidemiology and Biostatistics, and Department of Political Science, McMaster University, 1200 Main St. West, HSC-2D3, Hamilton, ON, Canada, L8N 3Z5; 2 Norwegian Knowledge Centre for the Health Services, P.O. Box 7004, St. Olavs plass, N-0130 Oslo, Norway; 3 Health Research Methodology PhD Program and Department of Clinical Epidemiology and Biostatistics, 1200 Main St. West, HSC-2D1 Area, Hamilton, ON, Canada, L8N 3Z5; 4 Norwegian Knowledge Centre for the Health Services, P.O. Box 7004, St. Olavs plass, N-0130 Oslo, Norway; Health Systems Research Unit, Medical Research Council of South Africa; 5 The Alfred Hospital, Monash University, Level 4, 89 Commercial Rd, Melbourne, VIC, Australia 3004; 6 Norwegian Knowledge Centre for the Health Services, P.O. Box 7004, St. Olavs plass, N-0130 Oslo, Norway; Section for International Health, Institute of General Practice and Community Medicine, Faculty of Medicine, University of Oslo, Norway

## Abstract

*This article is part of a series written for people responsible for making decisions about health policies and programmes and for those who support these decision makers*.

Differences between health systems may often result in a policy or programme option that is used in one setting not being feasible or acceptable in another. Or these differences may result in an option not working in the same way in another setting, or even achieving different impacts in another setting. A key challenge that policymakers and those supporting them must face is therefore the need to understand whether research evidence about an option can be applied to their setting. Systematic reviews make this task easier by summarising the evidence from studies conducted in a variety of different settings. Many systematic reviews, however, do not provide adequate descriptions of the features of the actual settings in which the original studies were conducted. In this article, we suggest questions to guide those assessing the applicability of the findings of a systematic review to a specific setting. These are: 1. Were the studies included in a systematic review conducted in the same setting or were the findings consistent across settings or time periods? 2. Are there important differences in on-the-ground realities and constraints that might substantially alter the feasibility and acceptability of an option? 3. Are there important differences in health system arrangements that may mean an option could not work in the same way? 4. Are there important differences in the baseline conditions that might yield different absolute effects even if the relative effectiveness was the same? 5. What insights can be drawn about options, implementation, and monitoring and evaluation? Even if there are reasonable grounds for concluding that the impacts of an option might differ in a specific setting, insights can almost always be drawn from a systematic review about possible options, as well as approaches to the implementation of options and to monitoring and evaluation.

## About STP

*This article is part of a series written for people responsible for making decisions about health policies and programmes and for those who support these decision makers. The series is intended to help such people ensure that their decisions are well-informed by the best available research evidence. The SUPPORT tools and the ways in which they can be used are described in more detail in the Introduction to this series *[1]. *A glossary for the entire series is attached to each article *(see Additional File 1). *Links to Spanish, Portuguese, French and Chinese translations of this series can be found on the SUPPORT website *http://www.support-collaboration.org. *Feedback about how to improve the tools in this series is welcome and should be sent to: *STP@nokc.no.

## Scenarios

*Scenario 1: You are a senior civil servant and will be submitting a brief report to the Minister regarding the evidence to support an option that has been provisionally selected to address a high-priority problem. You are concerned about whether the findings of a relevant high-quality systematic review that was used to make the selection are likely to be applicable to your specific setting, and you want to ensure that this issue has been assessed by your staff*.

*Scenario 2: You work in the Ministry of Health and are preparing a brief report about an option that is being considered to address a high-priority problem. All that you have been told is that the report should summarise the findings from the most relevant high-quality systematic review and assess the applicability of the findings to your setting*.

*Scenario 3: You work in an independent unit that supports the Ministry of Health in its use of evidence in policymaking. You are preparing a detailed research report for the Ministry of Health about what is known and not known about an option to address a high-priority problem. You have been told that policymakers have found a particular systematic review to be persuasive but you want guidance on how to assess whether the findings of the review are applicable to your setting*.

## Background

**Figure 1:**
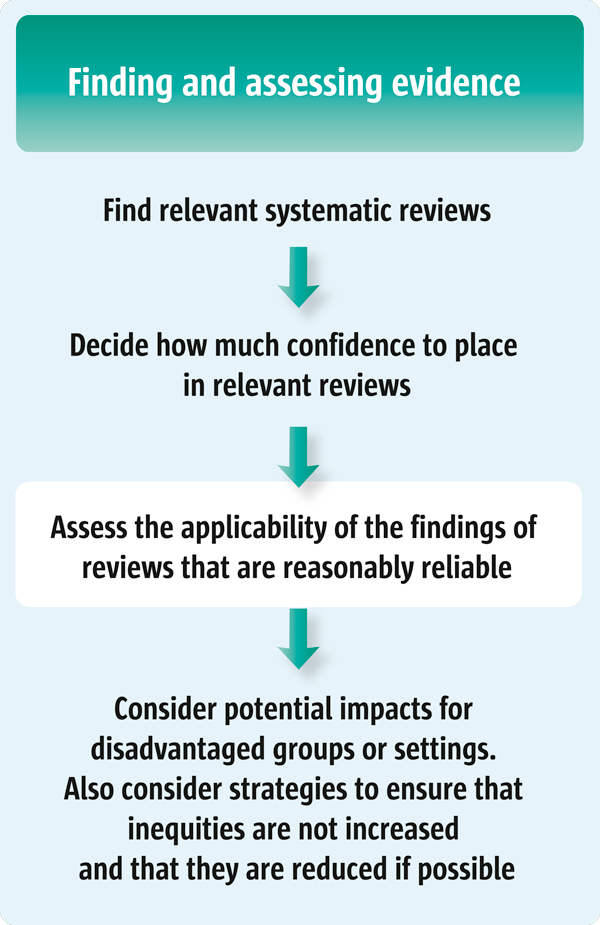
**Finding and assessing systematic reviews to inform policymaking**.

For policymakers (Scenario 1), this article suggests a number of questions that they might ask their staff to consider when preparing a brief report about a systematic review that could form the basis for selecting an option and communicating the rationale for the selection. For those who support policymakers (Scenarios 2 and 3), this article suggests a number of questions to guide the assessment of the applicability of the findings of a systematic review to a specific setting. This article is the third of four articles in this series about finding and assessing systematic reviews to inform policymaking (see also Articles 7, 8 and 10 [2-4]). Figure 1 outlines the steps involved in finding and assessing systematic reviews to inform policymaking.

Commonalities in human biology mean that a clinical procedure or drug will often work the same way in different patients. However, this is not always the case and questions have thus been developed to help healthcare providers to assess the applicability of research evidence to their patients [5]. Differences between health systems often mean that a policy or programme option being used in one setting may not be feasible or acceptable in another setting. These differences may also mean that an option may not work the same way in another setting or that it may achieve different impacts in another setting [6,7]. For example, the implementation of user fees failed to achieve consistent positive impacts in many sub-Saharan African countries compared to countries in other regions. In part, this was due to a number of contextual considerations, such as people's lack of familiarity with paying for public health services [8]. A key challenge that policymakers and those supporting them must face, therefore, is to determine whether research evidence about the impacts of an option are applicable to their setting.

Systematic reviews make this task easier by offering a single summary of studies from different settings. The word 'settings', in this instance, refers to political/country *jurisdictions *(e.g. Canada or Cameroon, or their constituent provinces). But settings can also include *sectors *(e.g. primary care or hospital care), and *locales *(e.g. urban or rural). Systematic reviews can also assist with the process of making judgements about the applicability of the evidence to specific settings by providing a framework and, when available, research evidence that can be used to identify those factors that are essential for an option to work - or that might modify its impacts. A systematic review of pharmaceutical policies (i.e. referencing pricing, other pricing, and purchasing polices for drugs), for example, provided a summary of the factors that could influence the impacts of reference pricing, as well as the rationale for each factor [9]. These factors included the equivalence of the drug, incentives, exemptions, drug availability, price levels, and electronic information systems.

Unfortunately, many systematic reviews do *not *do the following:

• Highlight the features of the settings in which studies were conducted, particularly those features that might modify the impacts of an option

• Provide a framework for identifying potential modifying factors, or

• Provide research evidence about modifying factors

In these cases, policy analytic articles or narrative reviews may provide more helpful frameworks that could be used to inform judgements about the applicability of the evidence in a systematic review.

A framework for understanding corruption in the health sector and its determinants, for example, highlighted how health system arrangements (e.g. governance arrangements that limit monopolies, require transparency, and support enforcement) and other factors, influenced 'opportunities' and 'pressure' to abuse, as well as the rationalisation for abuse, and how this in turn influenced the abuse of power for private gain [10]. But, ideally, a systematic review about the impacts of anti-corruption efforts would also have described the relevant health system arrangements in the settings where the studies were conducted. Those features that might influence opportunities and pressure to abuse and the rationalisation of such behaviour, would be of particular interest as they would allow the reader to link the determinants identified by the framework with the findings presented in the review.

Applicability considerations are equally, if not more important, for other types of systematic reviews. Reviews of administrative database studies and of community surveys can help to place problems in comparative perspective, for example, and reviews of observational studies can help to characterise an option's likely harms. Reviews of qualitative studies can assist in understanding the meanings that individuals or groups assign to particular problems, how and why particular options work, and stakeholder views about experiences with particular options. What follows in this article, however, is more focused on systematic reviews about the impacts of an option. That said, this may provide some help in assessing the local applicability of the findings of reviews of observational studies about harms, be supported by reviews about how and why particular options work, and give some insights into how to approach local applicability assessments of other types of reviews.

## Questions to consider

The following five questions can guide how to assess whether the findings from a systematic review are applicable to a specific setting.

1. Were the studies included in a systematic review conducted in the same setting or were the findings consistent across settings or time periods?

2. Are there important differences in on-the-ground realities and constraints that might substantially alter the feasibility and acceptability of an option?

3. Are there important differences in health system arrangements that may mean an option could not work in the same way?

4. Are there important differences in the baseline conditions that might yield different absolute effects even if the relative effectiveness was the same?

5. What insights can be drawn about options, implementation, and monitoring and evaluation?

### 1. Were the studies included in a systematic review conducted in the same setting or were the findings consistent across settings or time periods?

If the studies included in a systematic review were conducted in the *same *setting where policymakers are based, or else in very *similar *settings, there may be little reason to be concerned about the applicability of the findings. Similarly, if the findings have been shown to be consistent across settings or time periods then similar impacts might be expected. On-the-ground realities and constraints, health system arrangements and baseline conditions, are likely to differ across settings and change over time, so consistent findings in these circumstances are likely to mean the findings are broadly applicable. (These three issues are the focus of the next three questions that follow in this section.)

The following information in systematic reviews can be used by policymakers to inform judgements related to such issues:

• Information about the settings of studies and specifications regarding the time periods over which the studies were conducted. This can typically be found in a section of the review entitled 'Characteristics of included studies' (or similar)

• Information about the consistency of findings can typically be found in the review abstract or in its 'Results' section

When information about settings and time periods is lacking in a systematic review, policymakers and those who support them could contact the authors of the review to see if they have this information and did identify key local applicability considerations. If this contact yields little of value, they could then retrieve the original studies to locate this information if the issue is of sufficiently high priority, and if resources and time allow. A potential benefit of the direct contact with review authors is that it may encourage them to give attention to information needed for local applicability assessments and considerations in future reviews.

Research comparing mortality rates in not-for-profit hospitals with mortality rates in for-profit hospitals provides an example of how such data can be used [11]. This research had been conducted over several decades in the United States during which the health system had changed dramatically. The research demonstrated remarkable consistency over time in the significant survival advantage of being treated in not-for-profit hospitals. Based on these findings, a policymaker from Canada might then conclude that a similarly consistent finding would be seen in a Canadian setting. And this conclusion might lead them to avoid the introduction of for-profit hospitals into the current system which consists only of not-for-profit hospitals (or at least to avoid using health benefits as a justification for doing so).

### 2. Are there important differences in on-the-ground realities and constraints that might substantially alter the feasibility and acceptability of an option?

If the studies included in a systematic review were conducted in settings with largely similar resource and capacity constraints to the setting where the findings may be applied, *and *largely similar perspectives and political influence amongst health system stakeholders, policymakers might reasonably expect that an option would be both feasible and acceptable in their own setting. However, policymakers will rarely be able to find information about resource and capacity constraints and stakeholder influence in a systematic review. Instead, they will find a description of the option that was studied. Typically they will be sufficiently familiar with the resources, capacity, and stakeholder influence in their own setting to enable them to judge the feasibility and acceptability of the option.

Policymakers in a setting with very significant resource and capacity constraints will have to think twice about the feasibility of an option [12]. Some settings, for example, may face a shortage of nurse practitioners and therefore any option requiring a significant role for this category of healthcare provider might not be feasible in the short-term [13]. Similarly, some settings have such limited financial resources that an option shown to have significant impacts, such as artemisinin-based combination therapies (ACT) to treat malaria, might not be considered feasible on a large scale without significant donor support [14]. Some health systems may be too overstretched to accommodate an increase in demand that may accompany the introduction of conditional cash transfers (i.e. the provision of money to households on the condition that they comply with certain health and healthcare-seeking behaviours) [14,15]. Or settings may lack the capacity within governments or among managers, healthcare providers and consumers (i.e. healthcare recipients and citizens) to support the widespread use of a particular option. Audit and feedback (i.e. the provision of healthcare providers with data about their performance), for example, might not be feasible in settings where routinely collected data are unreliable.

In a setting in which stakeholders are opposed to an option and have significant influence on practice and policy, policymakers may have to assess the likely acceptability of an option particularly carefully. Healthcare provider associations, such as nursing associations, for example, may resist the introduction or expansion of a lay health worker programme if they perceive that the income or status of nurses might be affected [14,16]. Civil society organisations, too, may actively oppose changes that would reduce prescription drug use among consumers, particularly for life-sustaining drugs, and drugs that are important in treating chronic conditions [14,17]. Such changes could include the introduction of caps (i.e. consumers are reimbursed up to a set maximum number of prescriptions), co-insurance (i.e. consumers pay a percentage of the price of the prescription drug), and co-payments (i.e. consumers pay a fixed amount per prescription drug).

Significantly, many on-the-ground realities and constraints can be addressed over time. Nurse practitioner training programmes, for example, can be scaled up and donors can subsidise the cost of an expensive drug like ACT. Similarly, governments can improve the quality of routinely collected data, and healthcare provider associations and civil society organisations can become engaged in a series of negotiations or dialogues.

### 3. Are there important differences in health system arrangements that may mean an option could not work in the same way?

If the studies included in a systematic review were conducted in settings with *largely similar *health system arrangements to the setting where the findings may be applied, particularly those that might substantially alter the potential impacts of an option, policymakers might reasonably expect similar relative effectiveness in their setting. Deciding whether health system arrangements might alter an option's impacts requires an understanding of how *and *why an option might work. Within a systematic review, policymakers may find both a framework and research evidence that will identify those factors essential for an option to work - or that might modify its impacts. Policymakers may also find a summary of those features of the settings in which studies were conducted that might modify the impacts of an option.

If a systematic review does not provide the information necessary to determine whether particular health system arrangements might result in an option not working in the same way, policymakers could look for:

• Policy analytic articles or narrative reviews incorporating helpful frameworks that could be used to identify factors that might modify the impacts of an option, and

• Detailed descriptions of the health system arrangements, specifically those that might substantially alter the potential impacts of an option, in the settings where the studies were conducted

The European Observatory on Health Systems and Policies publishes, and periodically updates, profiles of the health systems of a large number of middle- and high-income countries. These 'Health in Transition' (HiT) profiles can be found online http://www.euro.who.int/observatory/hits/20020525_1 and downloaded free of charge. The Health Policy Monitor provides a searchable online database of key health system features in some of the same countries http://www.hpm.org/en/Search_for_Reforms/Search.html. Many World Health Organization regional offices also provide profiles of the health systems of countries in their region (e.g. http://www.searo.who.int/EN/Section313/Section1515_6038.htm).

Policymakers in a setting with very different health system arrangements, specifically arrangements that appear significant in determining whether an option will function in the same way, should be cautious about assuming that comparable relative effectiveness could be achieved. For example, in a review of reference drug pricing [9], six of the ten studies were conducted amongst older people/pensioners in British Columbia, Canada. Policymakers in other settings may well conclude that they will *not *be able to achieve comparable impacts to those seen in the Canadian example if they have any of the following issues within their own health system arrangements:

• Inadequate incentives for consumers, healthcare providers, pharmacists and pharmaceutical companies to comply with the reference drug price system, and

• An electronic processing system that lacks the capacity to realise the low administration costs associated with identifying, prescribing and dispensing the reference drugs and with handling exemptions

Similarly, other pricing policies examined in competitive pharmaceutical markets may yield a different relative effectiveness in markets with monopolies.

Unlike the possibility of associated change in on-the-ground realities that we discussed in Question 2 earlier, there is less chance that health system arrangements could be modified. Health system arrangements are difficult to change and typically the rationale underpinning a change would need to be more compelling than only the *possibility *that it would enhance the impact of a single option.

### 4. Are there important differences in the baseline conditions that might yield different absolute effects even if the relative effectiveness was the same?

If the studies included in a systematic review were conducted in settings with *largely similar *baseline conditions to those in which the findings may be applied, such as in terms of a programme's or policy's coverage of the population, policymakers might reasonably expect similar absolute effects in their setting (provided the answer they gave to Question 3 above led them to expect similar relative effectiveness). Policymakers will often be able to find information about baseline conditions within systematic reviews in a section titled 'Characteristics of included studies'. Alternatively, they may have to retrieve the original studies included in the review in the hope that baseline conditions were better described in them. Policymakers will typically be able to find local evidence about baseline conditions in their own setting. (Article 11 in this series addresses how to find and use local evidence [18].)

Policymakers in a setting with *different *baseline conditions may expect different absolute impacts. The absolute impact of audit and feedback, for example, is likely to be larger than in instances where the baseline compliance to recommended practice is low [19]. Similarly, the absolute impact of a pay-for-performance initiative may be larger in low- and middle-income countries (where small financial incentives may be larger relative to wages) than in high-income countries [20].

This question highlighting the link between baseline conditions and absolute effects is also highly relevant in clinical settings in which the relative effectiveness of a clinical intervention is often the same across patients but where patients' baseline risks may vary quite dramatically [21,22]. The question is also highly relevant in public health settings where immunisation programmes, for example, might be introduced in countries with very different baseline conditions. Article 16 of this series discusses the use of balance sheets to summarise important impacts and provides further detail about relative effectiveness and absolute impacts [23].

### 5. What insights can be drawn about options, implementation, and monitoring and evaluation?

Even if the findings from systematic reviews are not directly applicable to a given setting, important lessons can still be drawn. Policymakers may be provided with an idea for an option that they might otherwise not have considered. They may also gain insight into how options have been implemented in other settings. And they may be able to draw directly on the systematic review itself in developing a monitoring and evaluation plan. Policymakers may learn, for example, about a new approach to supporting team-based care, the importance of engaging both mid-level managers and front-line nurses in the implementation of an option, and what types of outputs and outcomes they should track as they monitor and evaluate the implementation of a selected option.

Table 1 and Table 2 provide examples of an assessment of the applicability of a systematic review.

**Table 1:** An assessment of the local applicability of a systematic review about home care (from the perspective of a Canadian policymaker)

Policymakers assessing the applicability of a 2005 review of home care could apply the series of questions discussed earlier as follows [24]:


1. Were the studies included in the systematic review conducted in the same setting or were the findings consistent across settings or time periods?
• 22 studies were included in the review
◦ 9 from the United Kingdom (UK)
◦ 3 from Australia
◦ 1 each from Italy, Norway, and the United States
◦ 7 were not described in a way that identified the country in which the study was conducted
• Findings were not consistent across settings
• Two studies were published in 1978 while the others were published from 1992 onwards. Many did not specify a time period, making it difficult to support the contention that the findings were consistent over time periods

2. Are there important differences in on-the-ground realities and constraints that might substantially alter the feasibility and acceptability of an option?
• In Canada, nurses are in tremendous demand (particularly in hospitals) and many are not used to the scope of practice required in home care settings. This means that many nurses might not embrace career opportunities in home care settings
• In Canada, unlike in the UK where 9 of 13 identifiable studies were conducted, citizens differ in whether they have supplementary coverage permitting more intensive home care. This means that relatively more wealthy people may get access to home care than the less well off
• In Canada, unlike in the UK, home care recipients and their families may have to travel very long distances if they have to seek acute care. Some may therefore delay their discharge from hospital; others may suffer if a hospital transfer is difficult
• In Canada, nurses may face a drop in pay if they move from hospitals to the community. Many of them may therefore actively oppose a shift from hospital care to home care
• In Canada, there is even more of a separation between health and social services (at least outside the province of Quebec) than there is in the UK, which means that caregivers may face a greater burden that is not covered by social services

3. Are there important differences in health system arrangements that may mean an option could not work in the same way?
• In Canada, as suggested earlier, home care recipients and their families cannot rely on the same breadth of services available to those in the UK (at least outside the province of Quebec)
• In Canada, unlike in the UK, there is a governmental commitment to first-dollar coverage for hospital-based and physician-provided care but not for home care, which means that Canadian home care recipients and their families may face significant financial barriers to accessing home care
• In Canada, unlike in the UK, most Canadians are not 'attached' to a multi-disciplinary primary healthcare practice, and some Canadian home care recipients would not even have a regular primary healthcare provider

4. Are there important differences in the baseline conditions that might yield different absolute effects - even if relative effectiveness was the same?
• In Canada, home care is already well established for most types of care, which means that the benefits may be small in absolute terms, at least for those not facing financial barriers

5. What insights can be drawn about options, implementation, and monitoring and evaluation?
• In Canada, admission-avoidance schemes may be a relatively unknown option compared to well-established schemes, such as the early discharge of elderly medical patients, or patients following surgery, or care of terminally ill patients
The review has now been updated and divided into two separate reviews, one of which deals specifically with admission-avoidance schemes and would be particularly relevant to Canada [25].

**Table 2:** An assessment of the local applicability of a systematic review on lay health worker interventions (from the perspective of a South African policymaker)

Policymakers assessing the applicability of a 2006 review of lay health worker (LHW) interventions for maternal and child health in primary and community healthcare could apply the following series of questions [26,27]:
1. Were the studies included in the systematic review conducted in the same setting or were the findings consistent across settings or time periods?
• 48 studies were included in the review
◦ 25 from the Unites States (US)
◦ 3 from the United Kingdom (UK)
◦ 2 each from Brazil, South Africa and Tanzania
◦ 1 each from Bangladesh, Canada, Ethiopia, Ghana, India, Ireland, Mexico, Nepal, New Zealand, Pakistan, Philippines, Thailand, Turkey, and Vietnam
• Findings were not always consistent across settings
• Most studies were published from 1995 onwards although one study was published in 1980. It is not clear from the review whether the findings were consistent over time periods

2. Are there important differences in on-the-ground realities and constraints that might substantially alter the feasibility and acceptability of an option?
• In South Africa, concerns have been expressed about the capacity of the health system and non-government organisations (NGOs) to provide clinical and managerial support for a very large scale-up of LHW programmes, particularly in currently under-resourced areas where, it could be argued, they are most needed. Capacity may be different from the high-income settings (US, UK) in which many of the studies were conducted
• In South Africa, there is some resistance among nurses, and within nursing professional associations, to extending the scope of practice of LHWs. This may restrict the range of tasks that LHWs are able to take on. While the acceptability of LHWs to consumers seems reasonable, based on observations from existing programmes, this is likely to vary across settings in the country and for different tasks (e.g. immunisation, breastfeeding promotion)
• In South Africa, most LHWs are currently involved in providing home-based care to people living with HIV/AIDS and treatment support to this group and to people with TB. It is not clear how feasible it would be to extend their roles to include the areas shown to be effective in the review (immunisation promotion, treatment of childhood infections, breastfeeding promotion). Furthermore, the LHW interventions shown to be effective in the review were focused on very specific health issues, such as the promotion of breastfeeding or immunisation uptake. Little evidence was identified regarding the effectiveness of more 'generalist' LHWs who are given responsibility for delivering a range of primary healthcare interventions
• In South Africa, norms and traditions regarding breastfeeding as well as differing baseline levels of breastfeeding and high rates of HIV/AIDS among mothers may alter the applicability of the review findings on LHWs for breastfeeding promotion

3. Are there important differences in health system arrangements that may mean an option could not work in the same way?
• In South Africa, LHWs are not licensed to dispense antibiotics for the treatment of acute respiratory infections in children or to dispense anti-malarial drugs. It may therefore be difficult in the short- to medium-term to extend their scope of practice in this way, even if shown to be effective in a review
• In South Africa, most LHWs are employed by NGOs, who receive funding from the government for the LHWs' salaries. It is not clear how secure this funding mechanism is

4. Are there important differences in the baseline conditions that might yield different absolute effects - even if relative effectiveness were the same?
• Baseline immunisation rates may be lower in South Africa than in some of the settings where the studies on LHWs for immunisation were conducted (Ireland, USA). Higher absolute effects might therefore be anticipated in South Africa

5. What insights can be drawn about options, implementation, and monitoring and evaluation?
• Most of the LHW interventions shown to be effective were focused on single tasks. The effectiveness of 'generalist' LHWs who deliver a range of primary healthcare interventions needs evaluation.

## Conclusion

Assessments of the applicability of the findings of a systematic review can take a lot of time to do well. Such assessments are critical, however, when an option is being proposed on the basis of a relevant high-quality systematic review. Policymakers and other stakeholders need to know whether they can expect similar findings in their own settings. Unlike an assessment of the quality of a review, which can often be delegated to researchers, a local applicability assessment must be done by individuals with a very good understanding of on-the-ground realities and constraints, health system arrangements, and the baseline conditions in the specific setting. The assessment of local applicability is a domain in which policymakers and those who support them need to be actively engaged.

## Resources

### Useful documents and further reading

- Dans AL, Dans LF, Guyatt GH: **Applying results to individual patients**. In *Users' Guides to the Medical Literature. A Manual for Evidence-Based Clinical Practice*. Edited by Guyatt GH, Rennie D, Meade MO, Cook DJ. New York, USA: McGraw Hill; 2008.

- Haynes RB: **Can it work? Does it work? Is it worth it?: The testing of healthcare interventions is evolving, **http://www.ncbi.nlm.nih.gov/pmc/articles/PMC1116525/. *BMJ *1999, **1999: **652-653.

### Links to websites

- SUPPORT Collaboration: http://www.support-collaboration.org - Example of a source of policymaker-friendly summaries of systematic reviews that provides an assessment of the applicability of the findings of each review (in this case to low- and middle-income countries), and that highlights the factors that policymakers need to bear in mind when assessing the applicability of the findings to their own setting.

- European Observatory on Health Systems and Policies: http://www.euro.who.int/observatory/hits/20020525_1 - Example of a source of (Health in Transition) profiles of the health systems of a large number of middle- and high-income countries.

- Health Policy Monitor: http://www.hpm.org/en/Search_for_Reforms/Search.html - Searchable online database of key health system features in a number of middle- and high-income countries.

## Competing interests

The authors declare that they have no competing interests.

## Authors' contributions

JNL prepared the first draft of this article. ADO, NMS, SL, RLG and AF contributed to drafting and revising it.

## Acknowledgements

Please see the Introduction to this series for acknowledgements of funders and contributors. In addition, we would like to acknowledge Sara Bennett, Mike Kelly, and staff in the Ontario Ministry of Health and Long-Term Care (MoHLTC) Planning Unit for helpful comments on an earlier version of this Article.

This article has been published as part of *Health Research Policy and Systems *Volume 7 Supplement 1, 2009: SUPPORT Tools for evidence-informed health Policymaking (STP). The full contents of the supplement are available online at http://www.health-policy-systems.com/content/7/S1.

## Supplementary Material

Additional file 1 Glossary Click here for file
